# Abstracts and Gleanings

**Published:** 1884-07-20

**Authors:** 


### ABSTRACTS AND GLEANINGS.

**Retained Placenta.**—The following instructive case was presented by Dr. T. R. Trotter to the last meeting of the Mississippi State Medical Association:

"I was called to see Mrs. B., Feb. 19th, and, upon inquiry, obtained the following history: She was about six and a half months advanced in pregnancy, and had aborted about eight days prior to my visit. The midwife who attended her failed to remove the placenta, assuring the family that it would pass off without any trouble. I learned that she had complained of great distress in the back and hypogastric region, and had more or less fever. The family, however, were resting easy, under the delusion that everything would be right in a few days, and failed to appreciate, until the morning of my visit, the great danger to which she was exposed. Upon attempting to arise from her bed, she was taken with a terrible flooding. I was at once sent for, seven miles, and when I arrived found her very restless, pulse almost imperceptible, extremities cold, and the whole surface bathed in a cold, clammy perspiration, a continuous flow of blood from the vagina, eyes sunken, and the whole countenance denoting the greatest anxiety.

I gave her at once brandy, and had her rubbed with dry mustard, and gave her a large dose of ergot, and attempted to introduce my hand into the uterine cavity to remove the placenta; this I failed to do, on account of its contracted condition. I, however, succeeded in introducing the index finger, and proceeded, as cautiously as possible, to remove a portion of the placenta; the remainder I could not remove because the finger could not be passed around it. The flooding moderated at once, but she was pale as a corpse, and passed from one fainting spell into another for several hours, notwithstanding the frequent administration of brandy, chicken tea, etc. In eight or ten hours she was seized with a severe rigor, great thirst, restlessness, insomnia, and a small, rapid pulse, and complained of soreness and pain in the region of the womb and intense pain in the back, so much so that she could remain in one position but a few moments. I gave her enemas of laudanum for the restlessness and pain, and spirits of nitre and gelseminum for the febrile stage. As soon as the sweating stage set in, I gave quinine, iron and arom. sulph. acid. I continued this treatment about six days. After that I gave quinine, iron and nutritious diet. I used, from the beginning, flannels wrung out of hot water, vinegar and laudanum, alternated with hot turpentine stupes to her bowels, frequently injecting the vagina with a solution of chlorinated soda.

The discharges per vaginam were very offensive, her tongue was very dry and red, and great tympanites and dysuria. To relieve this condition, I gave her turpentine internally and used it externally over the bowels.

About the thirteenth day abscesses were observed about her ankles, which, after being opened, gave issue to very offensive, purulent matter. About the sixteenth day she began to show unmistakable signs of improvement, and in twenty days after my first visit I considered her out of danger.

This was certainly a very unpromising case, the immense loss of blood, followed by the long continued putrid discharge, toxæmic fever, with its small, thready pulse, great prostration, tympanites, dysuria, and the putrid abscesses, presenting an array of complications sufficient to discourage the most sanguine.

I have reported this case to show what dire calamities ignorance or carelessness on the part of an accoucheur may bring upon an unsuspecting woman, and to impress upon the minds of the young members of the profession the great importance of seeing that every portion of the placenta is removed, and that the uterus is well contracted before leaving the patient.

I would not, however, advise any very prolonged or harsh measures to remove the placenta in cases of abortion prior to the fourth or fifth month, as by so doing the parts may be irritated and her suffering much increased. When the finger cannot be passed around the placenta, I always plug, if I am compelled to leave. and request the family to let me know should anything occur of an alarming character. In twelve to twenty-four hours I always return and remove the plug, and generally find the placenta and a few coagula of blood to come away with it.—*Medical and Surgical Journal.*

**Calomel an Instantaneous Nervous Sedative.**—Dr. Hatchett (in South. Clinic) says: From a close observation of its action, I am more and more impressed with the belief that the remedial effects of calomel are often *instantaneous;* that it is a prompt and powerful nervous sedative, commencing its action as soon as it reaches the mucous membrane of the stomach. This may appear incredible to some, but why should it? If it acts on the nervous system at all, why should it not act instantaneously? We must remember, it is the province of the nervous structure to convey impressions with electric speed; that it is composed of innumerable telegraphic filaments with their extremities ending and forming a complete network through the entire mucous membrane; and then it cannot appear so wonderful or incredible that calomel should instantly convey a sedative impression in cases of nervous excitement.

About the year 1869, I was called at night to see a lad of about twelve years of age, who had been having convulsions continuously from ten o'clock a. m. to nine p. m. The intervals between the convulsions had continued to grow less until, when I arrived, there was hardly any interval at all. His father, meeting me at the door, said, " You have come too late, doctor. My son is dying." And, really, it seemed so.

In the hope that some portion of it might reach the stomach, I poured a teaspoonful of dry calomel into his mouth, a good deal of which was blown out by his spasmodic breathing. I then mixed half a teaspoonful with syrup and placed it in his mouth. Within five minutes—I am sure it did not exceed that limit—his father said, "He is more quiet than he has been to-day." A lady friend, standing by, said, "Yes, more so than I have seen him." I sat by him and witnessed the gradually subsiding spasms until, after the lapse of thirty minutes, he sank into a composed sleep.

I visited him the next morning. He had rested well. After a dose of oil, he had two moderate evacuations from the bowels. Without further treatment, he continued to do well, and the recovery was complete.

If that were an isolated case, I should regard it now, as I did then, as a coincidence—that the disease had reached its culminating point and was on the eve of its departure, which would, probably, have been made with equal celerity had no remedy been given. My subsequent experience, however, has very positively impressed me with the belief that, in the case related, calomel is fairly entitled to credit for the cure. My subsequent readings, also, have helped much to confirm my impressions.

**Caffein in Therapeutic Doses.**—1. Caffein is generally borne better than digitalis and, when begun in small doses, is free from the unpleasant effects of the latter.

2. It regulates the heart, slows its rate and increases its working capacity.

3. It is more or less diuretic.

4. It is not only a substitute for digitalis, but in dangerous cases its effects are quicker and more certain.

5. The administration must be commenced with a small dose, not more than three grains, to test the susceptibility of the patient, then increased to eight or ten grains. It is useless to exceed twenty grains.

6. It may be used in all cases of heart disease in which digitalis is indicated, given with or without the latter.

7. In pyrexia caffein seems to reduce the temperature; at any rate, it is very useful in such cases as a heart tonic. It is often very beneficial in albuminuria.

9. Finally, it seems to stimulate the muscular walls of the intestine in strangulated hernia.—*Lyons Med.*—*N. E. Medical Monthly.*

**Placenta Prævia.**—Dr. John Reid reports a case of placenta prævia in the British Medical Journal, which terminatod rather differently from what is generally supposed to be the rule. Having intentionally lacerated the placenta to some extent, with the result of but covering his fingers with blood, he ruptured the membranes, when only some blood-streaked liquor amnii escaped. Within three hours the labor terminated naturally. The placenta was soon expelled, but the uterus still felt like a large, doughy mass; but soon after the expulsion of about a pint of blood, firm contraction of the uterus resulted, and the case progressed favorably. This is one of the many cases which militate against Simpson's theory of hemorrhage in placenta prævia, and may also add some additional force to the objections against meddlesome midwifery.—*Med. and Surg. Journal.*

**The Hypodermic Injection of Piles.**—The Gazette for April contains several answers to the query of our Tennessee brother as to the hypodermic mode of treating piles. I quite fully agree with Dr. Chase, of Geneseo, Ill., in his opinion as to why the carbolic acid treatment (or, more properly, the hypodermic treatment) has been cried down. I am not so orthodox that I cannot accept a good thing, regardless of from whence it came. I watched closely the results obtained by some traveling advertising doctors, and am free to confess that they cured cases of piles that I and other M. D.'s have totally failed to cure with the old modes of treatment. Well, like Mohamed, I went to the mountain, i. e., I bought from one of the so-called quacks—who, by the way, was a regular graduate from Rush College, Chicago—the secret, and have not been sorry that I did so. I have used it during the last eight years, without a failure or bad result in any of the many cases treated. I use carbolic acid (full strength) one part; sweet oil, two parts. Mix and inject into the pile tumor from one to four drops, according to the size of the tumor treated. I find it much more satisfactory to inject small quantities of the mixture, even if two or more injections are required into the same tumor, as the small injection does not cause very great soreness of the parts, as is the case wherein ten to twenty drops of a stronger mixture is used. When using the smaller injection, I sometimes find it necessary, after eight to ten days, to inject the tumor the second, and in rare instances the third time. The patient attends to his usual occupation during treatment, which he is not able to do when the larger and stronger injection is used.

The above facts will, doubtless, go a long way toward explaining why the fears of some of our physicians have been aroused. I think that danger from embolism can only arise, if it occur at all, when very large, strong injections are used. I am credibly informed that the treatment of piles, by injection of carbolic acid and oil, originated in the fertile brain of a doctor in the Southern army, during the late war, and that said doctor was a Tennesseean. —*Dr. Floyd Clendenen, in Ther. Gazette.*

**Exit National Board of Health.**—The abolition of the National Board of Health, which is provided for in the sundry civil bill, has been foreshadowed for some months past. In fact, for about a year the Board has had only a nominal existence, having no funds for the continuance of the scientific investigations previously forming so important a part of its functions, and its quarantine duties having been transferred to the Marine Hospital service. The circumstances which have led to this result have been of a comparatively insignificant character, but have been exaggerated by envious and designing persons into unpardonable offenses. Chief among them were: 1st, the dispute which arose between the Board and the Louisiana Board, and which, although the former has been fully justified in its course by the able defense of Dr. Cabell in the last annual report of the National Board, has nevertheless had great weight; 2nd, the charge of extravagance, which seems to rest chiefly upon the payment of three hundred dollars to a member of the Board (Dr. Verdi, a Homœopathic practitioner, of Washington) for an essay of eight and a half pages, three and a half of which are said to have been taken from an editorial in the London Times; 3rd, the claim that the Board is unnecessary, since all its duties can be discharged by another department of the service already equipped and provided, and like itself, a bureau pendent upon the Treasury Department, viz., the Marine Hospital Service.

The Marine Hospital Service is, in many respects, adapted for the work of quarantine, but whether it can carry out this work efficiently in view of the duties already devolving upon it, and which seem to have exacted its entire resources, may be considered doubtful. No provision seems to have been made for the prosecution of scientific research, which, under the Board, gave promise of such beneficent results to this country and to the world. —*Maryland Med. Journal.*

**The Cholera Bacillus.**—Dr. Koch has sent a seventh report of his work to the German minister of the interior, dated Calcutta, April 4.

He says that, since his previous report, twenty more autopsies have been made upon victims of cholera, and that the dejections of eleven cholera patients have been examined. This makes the total number examined in India—of cadavers, forty-two; of cholera patients, twenty-eight. The last cases do not furnish anything new, but confirm the results of previous examinations.

Experiments are now going on with reference to the power of certain agents—such as sublimate, phenol, etc.—to destroy the cholera bacilli. These investigations are not yet completed; but it seems to have been established that dry heat kills the bacilli, and that they remain active a long time in moist media.

As the Medical Record remarks, the most important feature of Koch's last report is his account of the discovery of the bacillus in certain water tanks. He states that throughout India there are numerous tanks of water which the inhabitants use for washing, drinking and bathing. These tanks are not kept very clean, and latrines are often placed near them; so that they often become infected with excrementitious fluids. Now, it has often been noticed that small epidemics of cholera are apt to surround these tanks, the disease affecting those who have been using the water. A circular epidemic of this kind was investigated by the Commission. It was limited exclusively to a ring of huts about the tank, and affected about one hundred persons. The water of the tank was examined, and the same bacilli were found that were seen in the cholera dejections. After the subsidence of the epidemic, none of these organisms could be found in the tank. These facts are looked upon as strong arguments in favor of the specific character of the organism which Koch has discovered.—*Pop. Sci. News.*

**Cannabis Indica.**—Medicinally, I have cause to be thankful to Cannabis Indica. A relative of my own took it regularly for six months, with bromide of potassium, every night, with the effect of removing acute bodily torture and mental anguish completely for the night. Dr. Clouston, some years ago, recommended large doses of cannabis with bromide of potassium in mania. Although I have had no personal experience of its use in acute mania, in cases of melancholia, and, indeed, in all cases of mental depression with sleeplessness, I have found a valuable and almost certain ally in this drug. Half a drachm to a drachm of bromide, with a grain or two of the extract, or from twenty to thirty drops of its tincture, seldom fails in the object we have in view. It dulls the feelings of anxiety, relieves the depression and gives restfulness, if not sleep. Hyoscyamus may be usefully combined if there be any visceral pain or disturbance.

A woman, in acute melancholia, said to me, "Doctor, I must kill my children. Send me to an asylum." I answered, "Will you promise not to kill them until I have tried the effect of one medicine upon you?" She thought she could promise. A drachm of bromide of potassium, with half a drachm of tincture of cannabis, two or three times a day, in a fortnight caused so much improvement, and even cheerfulness, that she declared she could now see the window open without the almost irresistible desire to throw her children down on to the pavement, which had possession of her whole mind before. My usual dose is a grain of the extract, or from twenty to thirty minims of its tincture.—*Brit. Medical Journal.*

**Chronic Dyspepsia.**—Dr. O. F. Taylor says (in the Atlantic Journal of Medicine): "I wish to call attention to listerine for chronic dyspepsia.

"W. H., age thirty-five, came to me for treatment of dyspepsia of long standing. His pain and discomfort was great, the stubbornness of the disease having produced mental depression and attendant evils. I put him on the following prescription: Listerine, two oz.; water, 4 oz.; one teaspoonful before meals After several weeks he returned much improved, and reported he was able to retain everything he ate with very little intestinal pain except when tempted to take heavy diet, such as fat meats, cabbage, &c. I then prescribed listerine, 4 oz.; water, 4 oz.; to be taken as before, and he has since obtained complete relief, and is regaining flesh and spirits without further treatment.

"I have had several similar cases of stomach derangement, and the listerine certainly gives me better results than any other remedy, and I would not be without it. It controls acidity promptly, produces a cooling and pleasing sensation, and is grateful generally to a patient thus afflicted."

Apropos of the above, we prescribed, ℞ Listerine, 2 ℥. S. Teaspoonful after meals in water, for a case of chronic intestinal dyspepsia with very gratifying results.

It is true the patient, an old lady, was not permanently cured of her trouble, yet the fermentation of starchy and saccharine food was arrested; consequently, gaseous distension of the bowels and colic, which before had caused her painful and sleepless nights, were followed by ease and sound sleep upon taking listerine.

**Carbolic Acid Injections of Piles.**—In the March No. of the Gazette appears a letter of enquiry from Dr. Grirard, of Winchester, Tenn., about the strength of carbolic acid injections in hæmorrhoids. I have been using the hypodermic treatment for piles for the past four or five years, and with universal success. The second case I had was a physician from Middlesex county, Virginia. He came to Brooklyn to consult some eminent physician in New York for piles and prolapsus of the rectum. I remarked to him, when he made known his business, that he need not go any further than my house—I could relieve him without the knife, ligature, ointments or caustic, and the operation would not interfere with his outdoor movements, as it produced only temporary pain. I attack only one pile daily, and where there are five or six piles, it sometimes happens they are relieved by injecting every alternate one; but it is safest to attend to each one in turn, and thus effect the entire removal of all. In this doctor's case he had had a bad prolapsus of the rectum, and had used sundry instruments and appliances to remedy his trouble, but none of them afforded him any relief. The rectum was promptly relieved at the last operation, and he returned home at the expiration of a week a sound man, so far as his piles and prolapsus of the rectum were concerned. He reported to me about twelve months afterward as still relieved, and no return whatever of hæmorrhoids or prolapsus. Let me say that in very large tumors it may be necessary to inject them two or three times before the circulation is cut off, as was the case recently with a friend of mine in Virginia. I vary my prescription a little, according to the case for treatment:

℞ Carbolic acid crystals........................... ʒ ij,

Pure glycerine.................................. ʒ ss,

Fluid ext. ergot (fresh)......................... ʒ iij.

M. and use in syringe from 5 to 6 minims at each injection.

I sometimes add a fractional part of a grain of tannin if there is not a prompt coagulum in the pile.—*Dr. Wm. T. Fleet in Ther. Gazette.*

**Alumen Ustum in Intermittent Fever.**—Schidowski, (Wratsch): Burnt alumen has long been known as a febrifuge. S—— does a large country practice, being alone in a district of 70,000 inhabitants, and he had only three pounds of quinine at his disposal for a whole year. He resorted to alum with good results. Two doses of eight grains each, one to three hours before the recurrence of the fever, effected the object. The powder is given dry and water is drunk copiously after it. He also saw enlargement of the spleen reduced by it.—*American Medical Digest, May 15.*

**Chloroform Narcosis During Sleep.**—Dr. John Gray, of Trufant, Mich., reports in the Record a case in which he administered, during sleep, to a child three years of age, chloroform to complete narcosis, during which a minor surgical operation was done. This is the second case of the kind reported during the year.—*Med. News.*

**Salicylic Acid a Cure for Tic Douloureux.**—We frequently meet in our practice cases of tic douloureux, that often so exceedingly painful neuralgia of the fifth nerve, where an operation seems to promise the only radical cure. If we hear of a remedy which is said to have the same effect as the surgical interference, we become doubtful; but if no less reliable an authority than Prof. Nussbaum (Munich, Ærztl. Intelligzbl., 38, 1883) assures us of the fact, our hope increases. Recently, a number of such cases had been sent to N. for the purpose of having the operation performed, and, after a number of carefully instituted experiments, this great surgeon recommends a trial with salicylic acid before proceeding to stretching or to resection of the nerve. In all the recently-sent cases he first tried this remedy, and he found it in every one a radical cure. Not only a palliative effect, but really an utter disappearance of the painful disease was the result in every case. Especially in cases of rheumatic nature, N. is positive of having discovered in salicylic acid a specific for tic douloureux. He administered the drug in the following manner:

℞ Acidi salicylici, 0.2 .......................... gr. $3\frac{1}{8}$,

Sodii salicylatis, 2.0 .......................... gr. 32.

M. ft. pulv.

Within twenty-four hours the patient takes from four to six of such powders.—*Med. and Surg. Reporter.*

**Sulphide of Calcium for Scabies.**—Dr. ("Brit. Med. Journal") says that sulphide of calcium, known in Poorlaw service as golden lotion, is more effectual in the treatment of itch than conventional sulphur ointment. It may be made by the following formula:

Flowers of sulphur .......................... 100 parts,
Quick-lime .......................... 200 parts,
Water .......................... 1,000 parts.

Boil the whole for some time, stirring occasionally until the substance become incorporated, allowing the liquid to cool, and decant into hermetically sealed bottles. It should not be made in a metal vessel.

It is applied as follows: The patient is first put into a warm bath; he is then painted with a brush dipped in the solution and placed in bed, either in blankets or a flannel nightgown. After a short time, owing to the deposit of sulphur, the patient's body is almost the color of a guinea. The beneficial effects are speedily manifested; the itching ceases, and, as a rule, in simple cases, after another warm bath, the patient may be discharged cured.—*Amer. Med. Digest*, May 15.

**Extirpation of Goitre by means of the Elastic Ligature.**—An Italian journal reports several cases in which enlargements of the thyroid body have been successfully removed by means of the elastic ligature. An incision is made in the skin in which the ligature is placed, the wound being disinfected and the ligature tightened daily.—*Med. News.*

**Treatment of Intermittent Fever.**—Regarding the treatment of intermittent fever, Prof. D. Beaumetz, of Paris, in the Detroit Lancet, says.

"I advise you to give your quinine in one dose three hours before the paroxysm. As to the dose, it should be variable according to the intensity of the fever, and you can give fifty to seventy-five centigrammes or one gram (from seven to fifteen grains) of chlorhydrate or sulphate of quinine. I cannot too strongly urge you, breaking the bonds of tradition which has given the preference to the sulphate, to use the chlorhydrate instead, a salt more soluble, more rich in quinine, and for that reason more active.

"When the individual has for a long time suffered from attacks of intermittent fever, and when, moreover, he continues to live in localities infected by malaria, grave modifications are often effected in the functions of certain viscera. The spleen becomes enormously hypertrophied, the liver is enlarged, the blood is profoundly altered and symptoms supervene whose aggregate constitutes the malarial cachexia. I shall not here describe this cachexia, but what I can assure you is, that the alkaloids of cinchona are impotent against it. Here you will often witness the triumph of arsenic, and hydro-therapy is of benefit. This last means is one of the most powerful resolvents in splenic and hepatic engorgements, and signal success has been obtained in these cases by douches of cold water directed over the spleen or liver. Here the tonic medication is indicated under all its forms, but all means of this kind are without avail if the individual does not submit to the hygienic treatment which enables him, under certain circumstances, to avoid the toxic action of the marsh miasm."

The hygienic treatment referred to consists in either removing the poison (by draining murshes) or removing from the poison to a non-malarious district; restorative dietary, of which wine should be a part; and the use of water that is free from any paludal contamination. As a prophylactic, he advises that the inhabitants of miasmatic districts be continually kept under the influence of Peruvian Bark.—*Med. World.*

**Drugs Influencing the Sexual Powers.**—Drugs supposed to have an influence over the sexual powers are reviewed in the Eclec. Med. Advocate by Dr. Jos. A. Hause. He concludes that all aphrodisiacs are uncertain at best. Cantharides is an irritant to the genito urinary system, rather than a stimulant to the virile powers. It is a very dangerous drug. Phosphorus is a much more direct and reliable sexual stimulant. Instances have been recorded where phosphorus caused the most consuming passion until death ensued from complete exhaustion. "A chemist having thrown out some of his refuse preparations, in which was some phosphorus, they were partly drunken by a drake, who immediately afterward commenced cohabiting with his female companion in the most furious manner, and continued to do so till he fell down dead."

"Ether will occasionally awaken the generative instinct when nothing else will; and cases are recorded where females, during labor, while under its influence, experience all the pleasures of amative indulgence." This occasional action of ether doubtless accounts for the suits brought against dentists on charges of violating the persons of female patients. Of course these acts are wholly imaginary on the part of the patients.

"Cannabis Indica is of great value in treating those conditions arising from functional derangement of the sexual apparatus, and is frequently given with the most beneficial results in spermatorrhœa, loss of power, etc." The doctor considers damiana unreliable, like all the other so called aphrodisiacs. He believes that it has been over estimated.—*The Med. World.*

**Bromides in Treatment of Neurotic Diarrhœa of Children.**—At a recent meeting of the Harveian Society of London, a report of which we find in the Medical Times of London, Dr. Lees called attention to a class of cases, not very uncommon in children, in which the main symptom was an irresistible impulse to defecation experienced almost immediately after taking food. Colic pain might or might not be present, but there was no sensation of weight at epigastrium, heartburn, flatulence or other symptom of dyspepsia. The motions were usually semi-solid, not often watery or slimy, and frequently contained undigested food. Usually a motion was passed almost immediately after every meal, and, perhaps, once or twice more during the twenty-four hours. Dr. Lees pointed out that these symptoms were evidently due to a hyper-peristalsis of the alimentary canal, without increase of secretion, the two factors of ordinary diarrhœa being here disassociated. Such increase of peristalsis was probably due to irritation of the vagus nerve, which supplies the excitor fibers to the intestine, the splanchnics conveying the inhibitory fibers. The proximity of the nucleus of the vagus to that of the trigeminus, in the medulla, indicated the possibility that this increased excitability of the intestine might in part be due to dental irritation, the cases in question usually occurring during the period of the second dentition. Believing in the purely neurotic origin of the symptoms, Dr. Lees had treated several cases with bromide of potassium simply, without opium or any astringent, and had obtained immediate success, even in cases which had persisted for several months. The diarrhœa was usually arrested in a few days, and occasionally the children became so costive that the medicine had to be discontinued. Four cases were narrated, also a similar case occurring in an adult, in all of which speedy relief was given by the bromide. In conclusion, it was remarked that individuals who suffered from these symptoms were often of a markedly neurotic temperament, timid and easily frightened.—*N. Y. Medical Journal.*

**Too Much Feeding of Infants.**—Dr. Little, in Journal of Obstetrics, remarks: "For twenty-one years I have been physician to the Rochester Orphan Asylum. Each of these years had witnessed deaths from enteric diseases until 1882. In the early summer of that year I said to the matron, feed your babes but once in three hours during the day, but give them water to drink as often as they will take it. The summer came and went, and when frost appeared I congratulated her on the good results of our plan. Not a child had died, and no serious case of diarrhœa had occurred.

"'Yes," said she, "but it seemed cruel to feed these babes but three times a day.'

"She had actually carried out my instructions to the letter, as she had misunderstood them. Instead of once in three hours, she thought I had said three times a day! On the following (that is, last) summer the plan was carried out, giving the infants food once in three or four hours, according to the age, during the day, and an additional meal in the night if the child awoke and would not be quieted with a simple drink of water."

**Humane Blistering.**—When we think how painful the results of blisters are to our patients, and yet how valuable they are, we will hail with pleasure the following suggestion of Dr. Samuel Stretton in the British Medical Journal:

In an illness about fifteen years ago, I myself suffered considerable distress following the application of a large blister (which, no doubt, was instrumental in saving my life); but since that time I have never directed a similar torture for others, though I obtain satisfactory results from the blistering process I do employ. I direct the surface requiring such counter-irritation to be well covered with annular blisters about the size of the human iris, cut from vesicating tissue with an ordinary gun-punch, the centre being extracted with a punch of small size. Once secured to the surface, and covered with cotton wool and bandage, these blisters require no further attention. The discomfort created is so slight that I never meet with the slightest resistance to their application.—*Med. and Surg. Rep.*

**Varicose Veins.**—A writer in the Eclectic Med. Journal says: "I told the patient I would see what I could do. The outcome of my seeing what I could do was to go to the woods and get the inner bark of the Quercus Rubra and Rhus Glabrum. I directed a strong decoction to be made and used as a wash twice a day, after which she was to apply the elastic bandage.

"Internally, I gave her fluid ext. hamamelis ʒj three times a day. She began to improve at once, and now calls herself entirely well. I don't know whether it was the Hamamelis or the wash that did the work, but I know that she had worn the elastic bandage for a long while, and received no benefit from it alone."

**Bursting of Coffin.**—A peculiar case is related in the British Medical Journal. Several days after a woman's burial it was concluded to hold a post mortem. While opening the lid, the coffin burst with a loud noise, one of the boards striking the police inspector and knocking him down. Dr. McDonald, the medical officer in attendance, fainted, and remained unconscious for some time, and has since died. Another physician who was present is lying seriously ill.—*Weekly Med. Rev.*

**Etherization by Rectum.**—It has been found that etherization can be produced by rectum. Ether is placed in a bottle, a rubber tube is attached to the neck of the bottle, and to the other end of the tube may be attached the vaginal nozzle of a Davidson syringe. The bowels should be moved by enema before the operation. When the patient is ready, place the bottle in hot water and introduce the nozzle into the rectum, thus conducting the vapor into the rectum, It is surprising how soon the patient is etherized. The struggling, sense of suffocation, etc., that are present by the inhalation method are absent. The stage of excitement is often absent. Nausea and vomiting are seldom present. The method is being freely tested in New York city, with generally favorable results thus far.—*Med. World.*

**Cod Liver Oil as a Surgical Dressing.**—A writer in Med. and Surg. Reporter says, in concluding a paper on the uses of cod liver oil in surgery: "Thus I might go on, citing over a hundred different cases in which the cod liver oil, with the addition of carbolic acid, was the only dressing applied, but will be content, for the present, with those above mentioned. In every case I was compelled to note the increased vitality.

"My experience with cod liver oil as a surgical dressing has led me to use it exclusively, believing it to be superior to the olive oil generally used. To those who may desire to test the virtues of this new dressing, let me suggest that they use the purest article to be found in the market."

**Eucalyptus.**—A writer (in Pacific Med. and Surg. Jour.) says of the effects of the fluid extract of eucalyptus globulus, after an experience of eight months in its use in the Marine Hospital, that he regards it as a diuretic of rare virtue, capable of being administered when other diuretics in common use are inadmissible. It is an aromatic tonic, and has notable restorative effects in low states of the system, as typhoid fever, typhoid diarrhœa and dysentery. In vesical catarrh its action is very beneficial. It relieves headache, apparently, as the saline cathartics do, by its diuretic action. It does not impair, but rather improves the appetite. As an external application in chronic ulcers it has great value.

**Iodine in Intermittent Fever.**—A few weeks ago, while looking over some old medical journals, my attention was attracted to an article on the "Treatment of Malaria with Iodine." The results of this treatment, as reported, were very favorable. I had scarcely finished the article when I was called to see a case of intermittent fever, of quotidian type. Patient had been sick for four days previous. I administered tinct. iodine, gtt. v, largely diluted, four times a day. After a few doses, patient began to improve, and has had no chills since—over a month ago.—*Dr. Cree, in Ther. Gazette.*

**Substitute for Opium.**—Dr. R. Bartholow claims that tannate of cannabine is the nearest approach to a substitute for opium yet proposed.

